# Trophoblast Retrieval from the Cervix (TRIC) Across Early Gestation: A Multi-Method Assessment of Extravillous Trophoblast Recovery

**DOI:** 10.3390/ijms27156641

**Published:** 2026-07-25

**Authors:** Kirim Hong, Hee Yeon Jang, Ji Eun Park, Sung Han Shim, Sung Shin Shim, You Jung Han, Soo Hyun Kim, Hee Jin Park, Dong Hyun Cha

**Affiliations:** 1 Department of Obstetrics and Gynecology, CHA Ilsan Medical Center, CHA University, Goyang-si 10414, Republic of Korea; rachelkh@chamc.co.kr; 2 Department of Biomedical Science, College of Life Science, CHA University, Seongnam-si 13488, Republic of Korea; jhyeon@chabio.com (H.Y.J.); shshim@cha.ac.kr (S.H.S.); 3 Center for Genome Diagnostics, CHA Biotech Inc., Seoul 06125, Republic of Korea; ditto6626@chabio.com; 4 Department of Obstetrics and Gynecology, CHA Gangnam Medical Center, CHA University, Seoul 06125, Republic of Korea; ogshinyss@chamc.co.kr (S.S.S.); hanyj1978@chamc.co.kr (Y.J.H.); soohyunkim@chamc.co.kr (S.H.K.); coolsome72@chamc.co.kr (H.J.P.)

**Keywords:** trophoblast retrieval from the cervix (TRIC), extravillous trophoblast, HLA-G, gestational age, placentation, trophoblast recovery, prenatal diagnosis, fluorescence-activated cell sorting (FACS)

## Abstract

Trophoblast retrieval from the cervix (TRIC) is a non-invasive approach that enables access to fetal-derived extravillous trophoblast (EVT) cells as early as the first trimester of pregnancy, offering a unique opportunity for early placental and fetal assessment. Although TRIC has been reported to be feasible between 5 and 20 weeks of gestation, systematic evaluation of EVT recovery across early gestation using complementary analytical approaches remains limited. This study aimed to provide a multi-method assessment of EVT recovery across early gestation. Endocervical samples were collected from 53 pregnant women between 5 and 19 weeks of gestation. EVT cells were isolated by immunomagnetic separation using human leukocyte antigen-G (HLA-G)-specific antibodies and assessed by β-hCG immunofluorescence, fluorescence in situ hybridization (FISH) and fluorescence-activated cell sorting (FACS). Immunofluorescence and FISH analyses showed no significant differences in β-hCG-positive or fetal cell proportions among gestational age groups, supporting stable TRIC-based recovery across early gestation. Exploratory FACS analyses identified HLA-G-positive trophoblast populations, including in samples obtained at 5 weeks of gestation. Because FACS analyses were performed in a subset of the cohort, these findings should be considered exploratory and require validation in larger cohort-wide studies. Overall, our findings support the feasibility of TRIC throughout early gestation and confirm the successful recovery of fetal-derived EVT cells from as early as 5 weeks of gestation. Further studies using larger cohorts and standardized quantitative approaches are required to more comprehensively characterize EVT recovery across early gestation and to optimize the application of TRIC in placental and prenatal research.

## 1. Introduction

The placenta is a highly specialized organ that plays a central role in maternal–fetal communication and is essential for normal fetal growth and pregnancy maintenance. Among placental cell populations, extravillous trophoblast (EVT) cells are responsible for invading the maternal decidua, uterine vasculature, and endometrial glands during early placentation, thereby establishing the maternal–fetal interface and ensuring adequate placental perfusion [1,2,3,4]. Abnormal EVT differentiation and invasion have been implicated in various pregnancy complications, including preeclampsia, fetal growth restriction, recurrent pregnancy loss, and implantation failure [2,3,5,6]. Consequently, EVT cells have attracted considerable interest not only as targets for prenatal genetic analysis but also as valuable tools for investigating placental biology and pregnancy-related disorders.

Trophoblast retrieval and isolation from the cervix (TRIC) has emerged as a promising non-invasive approach for accessing fetal genetic material during early pregnancy [7,8,9,10]. Conventional non-invasive prenatal testing (NIPT), which is widely used in current prenatal screening, relies on the analysis of cell-free fetal DNA (cffDNA) in maternal plasma [11,12]. Although NIPT has demonstrated high sensitivity and specificity for common aneuploidies, its diagnostic performance is limited by factors such as low fetal fraction, placental origin of cffDNA, and the potential influence of confined placental mosaicism [13,14,15,16]. In contrast, TRIC enables the direct isolation of intact EVT cells containing the complete fetal genome, thereby offering a more direct and potentially more accurate approach for early prenatal diagnosis [17]. This unique feature has generated increasing interest in TRIC as a potential platform for early prenatal diagnosis and placental assessment.

EVT cells originate from the trophoblast lineage of the placenta and play a critical role in early placentation through their invasive and migratory properties [8,18,19]. During early pregnancy, EVT cells differentiate and invade maternal tissues, including the decidua, uterine vasculature, and endometrial glands [8,19,20]. Through interstitial and endoglandular invasion pathways, EVT cells can enter the uterine cavity and subsequently migrate or be transported toward the cervix via uterine secretions [8,21]. This biological process provides the fundamental basis for TRIC, as EVT cells residing in the endocervical canal can be collected using minimally invasive techniques such as a cytobrush during a Papanicolaou-like procedure [7,22]. Previous histological and molecular studies have demonstrated the presence of EVT cells in the uterine cavity and cervix as early as 5 weeks of gestation, supporting the feasibility of this approach in early pregnancy [9,23,24].

The concept of retrieving trophoblast cells from the cervix has been explored since the 1970s; however, early attempts were limited by low cell yield and significant contamination with maternal cells [25,26]. Advances in molecular and immunological techniques have significantly improved the reliability of TRIC. In particular, immunomagnetic isolation using antibodies targeting human leukocyte antigen-G (HLA-G), a specific marker of EVT cells, has enabled the enrichment of trophoblast cells with high purity, typically exceeding 90–95% human chorionic gonadotropin (β-hCG) positivity [7,17,22,27,28]. These isolated cells have been shown to contain fetal genetic material and to exhibit EVT-specific phenotypic characteristics, allowing for downstream applications such as fluorescence in situ hybridization (FISH), polymerase chain reaction (PCR), and next-generation sequencing [17,18]. Recent studies have demonstrated the feasibility of TRIC for fetal genotyping, detection of chromosomal abnormalities, and analysis of single-gene disorders, highlighting its potential as a diagnostic tool [10,24,29,30].

Beyond its application in prenatal genetic diagnosis, TRIC has also emerged as a valuable platform for investigating placental biology and for the early prediction of pregnancy-related complications. EVT cells obtained from the endocervical canal have been shown to retain molecular and functional characteristics reflective of placental status, making them a potential surrogate for in vivo placental tissue during early gestation [7,8]. Several studies have demonstrated that protein expression profiles in TRIC-isolated EVT cells correlate with subsequent pregnancy outcomes. For example, differential expression of placental biomarkers such as pregnancy-associated plasma protein A (PAPP-A), placental growth factor (PlGF), soluble fms-like tyrosine kinase-1 (sFlt-1), and endoglin has been observed in EVT cells from pregnancies that later developed preeclampsia or fetal growth restriction [31,32]. In particular, Fritz et al. and Bolnick et al. reported that EVT cells collected between 5 and 19 weeks of gestation exhibit distinct biomarker signatures that precede the clinical onset of placental dysfunction, suggesting that TRIC may enable early risk stratification for adverse outcomes [31,33]. In addition, studies have shown that the number of EVT cells retrieved via TRIC may be reduced in abnormal pregnancies, such as early pregnancy loss or ectopic pregnancy, further supporting the association between trophoblast biology and pregnancy viability [23,33]. Moreover, TRIC allows access to trophoblast cells during a critical window of early placental development, when direct assessment of placental tissue is otherwise not feasible [8]. This unique capability positions TRIC as a potential tool for both early diagnosis and risk stratification in obstetrics.

Despite these advances, most previous studies have primarily focused on demonstrating the feasibility and technical optimization of EVT isolation, with relatively limited investigation into the quantitative dynamics of trophoblast yield across gestational age [7,8,22]. Although TRIC has been reported to be feasible from approximately 5 to 20 weeks of gestation, systematic evaluation of EVT recovery across early gestation has remained limited. Because the efficiency of trophoblast retrieval directly influences downstream molecular applications, a comprehensive understanding of EVT recovery throughout early gestation is important for optimizing the clinical application of TRIC.

Therefore, the aim of this study was to evaluate EVT recovery following TRIC across early gestation using complementary analytical approaches. Specifically, we investigated whether fetal trophoblast cells could be consistently recovered from early pregnancy, including as early as at 5 weeks of gestation.

## 2. Results

### 2.1. Gestational Age-Dependent Recovery of EVT Cells Following TRIC

#### 2.1.1. Baseline Characteristics Across Gestational-Age Groups

To evaluate trophoblast recovery at different pregnancy stages, 53 samples of pregnant women were categorized into three gestational age groups (5–7 weeks, 8–12 weeks, and 13–19 weeks). The three groups differed significantly in gestational age (a–c, *p* < 0.05), whereas maternal age, BMI, gravidity, and parity were comparable among groups (all *p* > 0.05) (Table 1, Supplementary Table S1). The data were calculated as mean ± standard deviation (SD).

**Figure 1:**
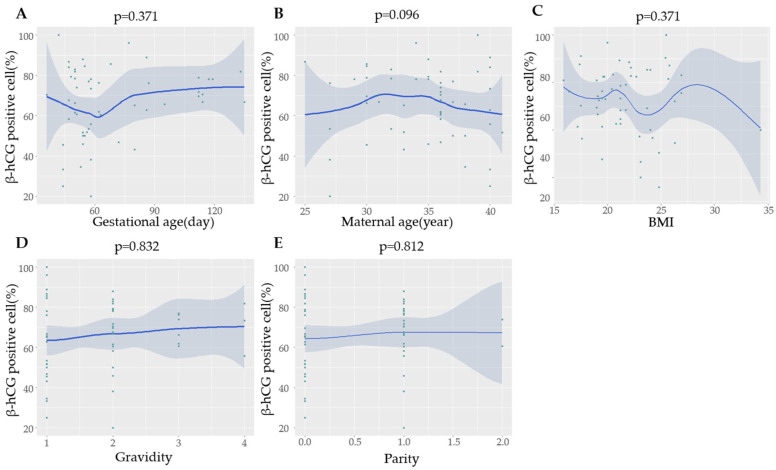
β-hCG-Positive Trophoblast Cell Proportion According to Gestational Age and Maternal Characteristics. (**A**) gestational age (days); (**B**) maternal age; (**C**) body mass index (BMI); (**D**) gravidity; and (**E**) parity. Green dot: individual samples; Blue line: fitted regression curve; Blue shaded area: 95% confidence interval.

No significant associations were observed between β-hCG positivity and gestational age, maternal age, BMI, gravidity, or parity (all *p* > 0.05) (Figure 1).

#### 2.1.2. Immunofluorescence-Based Quantification of EVT Cells

**Figure 2:**
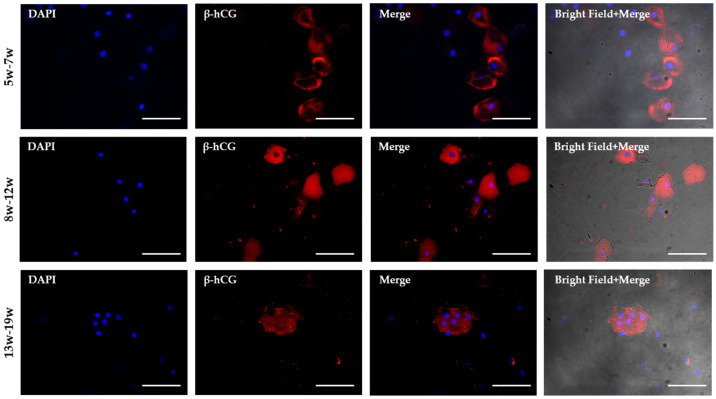
Analysis of fetal cell expression and cell count using immunofluorescence staining (scale bar = 75 μm).

Trophoblast retrieval was initially assessed by immunofluorescent quantification of β-hCG-positive cells. For immunofluorescence analysis, isolated HLA-G-positive cells were loaded onto glass slides at 100 cells per slide and analyzed by β-hCG immunostaining. All available non-overlapping microscopic fields on each slide were examined, with approximately 100 cells evaluated per slide. Only fetal-specific β-hCG-positive cells were included in the quantitative analysis. Image analysis and cell counting were performed in a blinded manner, without knowledge of gestational age. Representative immunofluorescence images demonstrated β-hCG-positive trophoblast cells across all gestational age groups. Trophoblast cells were identifiable as early as 5–7 weeks of gestation, and larger β-hCG-positive cell clusters were more frequently observed in samples obtained after 8 weeks (Figure 2).

**Figure 3:**
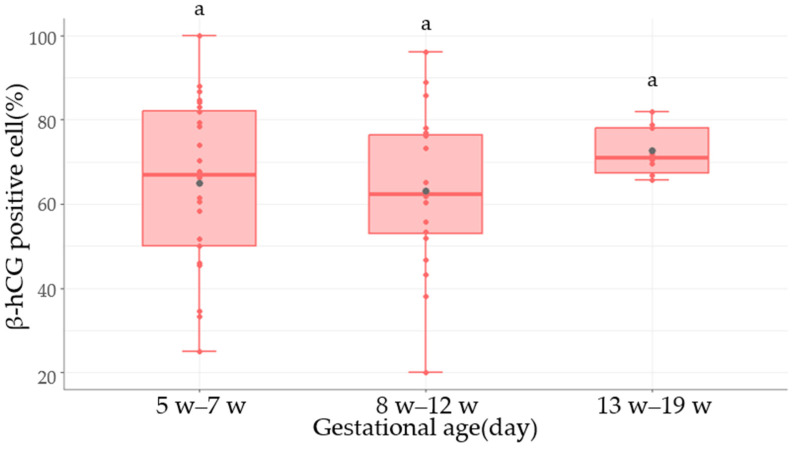
Proportion of Trophoblast and Fetal Cells across Gestational Age by Immunofluorescence. Red dots: individual samples; gray dots: mean values. ^a^ No significant difference among groups (*p* > 0.05).

The percentages of β-hCG-positive cells were calculated as mean ± standard error (SE). Immunofluorescence analysis demonstrated consistently high β-hCG positivity across all gestational age groups, ranging from 63.2% to 72.8% (Figure 3). Although the numerically highest mean β-hCG-positive rate was observed in the 13–19 weeks group, no statistically significant differences were detected among groups. These findings suggest that the proportion of β-hCG-positive trophoblast cells remained relatively stable across the evaluated gestational age range.

Notably, HLA-G-positive cell counts were significantly increased in the 8–12 week group compared with the 5–7 week group (*p* < 0.05), whereas significantly decreased counts were observed in the 13–19 week group compared with the 8–12 week group (*p* < 0.05). However, no statistically significant difference was observed between the 5–7 week and 13–19 week groups, which showed similar cell counts (Table 2, Supplementary Table S2).

#### 2.1.3. FISH-Based Assessment of Fetal Cell Proportions

**Figure 4:**
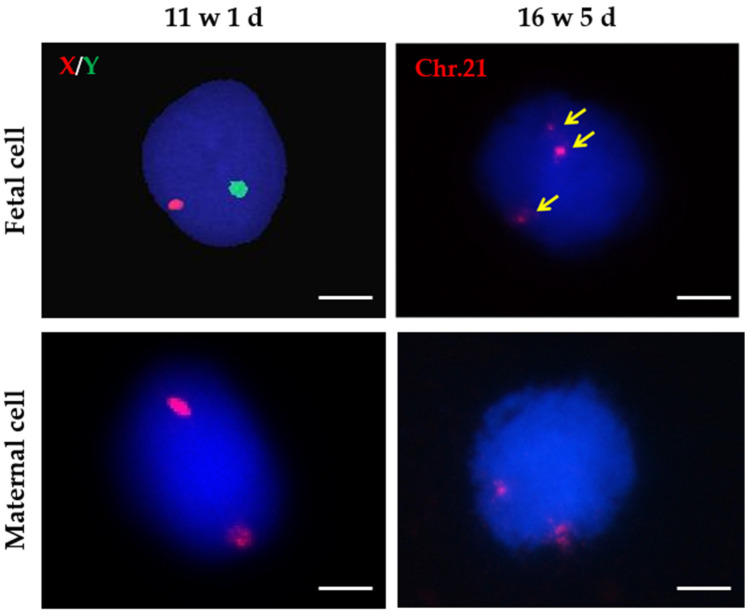
Representative FISH Images Confirming Fetal Cells Using Y-Chromosome and Chromosome 21 Probes (scale bar: 5 μm). Yellow arrow: chromosome 21.

For FISH analysis, fetal cells were identified using either Y-chromosome probes in male fetuses or chromosome 21 probes in fetuses with trisomy 21, allowing discrimination from maternal cervical cells. Representative FISH images are shown in Figure 4, illustrating the detection of Y chromosome signals in a male fetus at 11 weeks and 1 day of gestation and chromosome 21 signals in a fetus with trisomy 21 at 16 weeks and 5 days of gestation.

**Figure 5:**
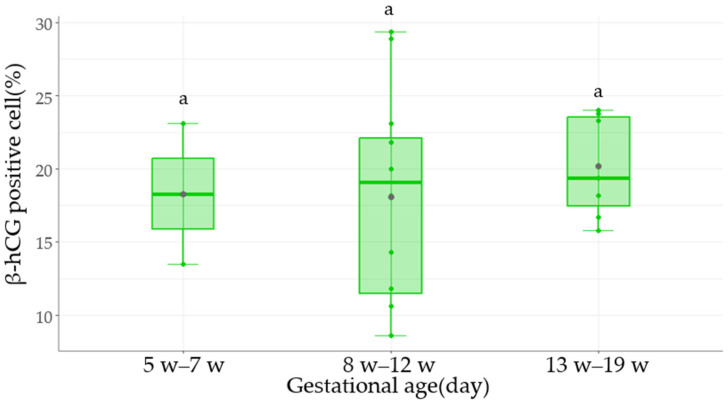
FISH-Based Fetal Cell Proportions across Gestational Age Groups. Green dots: individual samples; gray dots: mean values. ^a^ No significant difference among groups (*p* > 0.05).

FISH analyses were restricted to cases with unequivocal fetal cell identification based on Y-chromosome signals or confirmed chromosomal aneuploidies. Consequently, the number of samples included in each gestational age group differed slightly from that of the overall study cohort. The percentages of Y chromosome-positive cells and chromosome aneuploidy cells were quantified to estimate fetal cell rates, and values were expressed as mean ± SD (Table 3, Supplementary Table S3). Fetal cell rates detected by FISH showed a slight increase in the 13–19 weeks group, reaching 20.2%; however, the difference was not statistically significant (Table 3, Figure 5).

### 2.2. FACS-Based Assessment of HLA-G-Positive Trophoblast Populations

**Figure 6:**
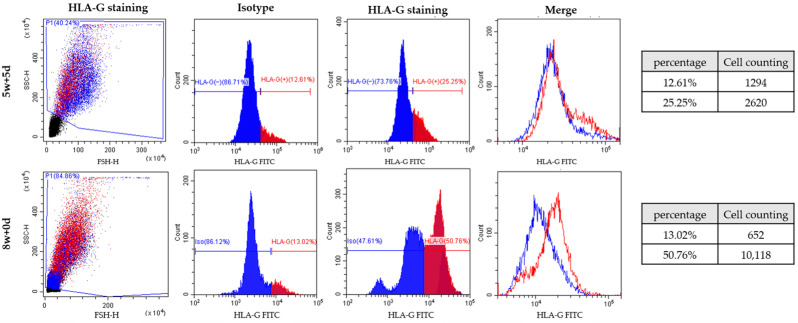
FACS-Based Quantification of HLA-G-Positive Trophoblast Cells at Different Gestational Ages.

Fetal trophoblast cells in cervical samples were additionally analyzed using fluorescence-activated cell sorting (FACS) (Figure 6). Compared with magnetic-activated cell sorting (MACS), FACS enabled more precise identification of HLA-G-positive cell populations based on fluorescence intensity and cell size. Initially, HLA-G-stained cells were gated using forward scatter (FSC) and side scatter (SSC) to define the target cell population (Supplementary Figure S1). The gated cell population increased from 40.23% at 5 weeks and 5 days of gestation to 84.86% at 8 weeks of gestation. Subsequently, HLA-G FITC fluorescence intensity was compared between isotype control and HLA-G-stained groups. At 5 weeks and 5 days of gestation, FITC-positive cells accounted for 25.25% of the HLA-G-stained group, compared with 12.61% in the control group. At 8 weeks of gestation, FITC-positive cells increased to 50.76% in the HLA-G-stained group, whereas the control group showed 13.02% positivity. These findings demonstrate that HLA-G-positive trophoblast cells can be detected as early as 5 weeks of gestation, although differences in positivity between the two representative samples should be interpreted with caution. Because comprehensive cohort-wide FACS analyses were not performed, these observations are exploratory and are intended to support the feasibility of fetal trophoblast identification rather than to establish gestational age-dependent changes in trophoblast recovery efficiency.

## 3. Discussion

### 3.1. Multi-Method Assessment of EVT Recovery Across Early Gestation

In this study, we performed a multi-method assessment of extravillous trophoblast (EVT) recovery following trophoblast retrieval and isolation from the cervix (TRIC) across early gestation. Our findings demonstrate that EVT retrieval is feasible from as early as 5 weeks of gestation and are consistent with previous reports showing that fetal-derived trophoblast cells can be successfully recovered from the endocervical canal during very early pregnancy. Immunofluorescence-based β-hCG positivity and FISH-based fetal cell proportions did not differ significantly among gestational age groups, supporting relatively stable trophoblast recovery across early gestation [9,34,35,36]. Exploratory FACS analyses identified HLA-G-positive trophoblast populations in selected samples, including those obtained at 5 weeks of gestation, confirming that these cells can be detected throughout early gestation. Because comprehensive cohort-wide FACS analyses were not performed, these observations should be interpreted as exploratory and hypothesis-generating rather than evidence of a reproducible gestational age-dependent effect [1,2,34,36]. Although the exploratory FACS observations may reflect biological differences in EVT populations during early placentation, technical and sampling-related explanations cannot be excluded. Therefore, systematic cohort-wide validation is required before any biological interpretation regarding gestational age-related differences can be made.

Interestingly, the trends observed by FACS were not fully reflected in either immunofluorescence or FISH analyses. Several explanations may account for this discrepancy. Immunofluorescence and FISH are microscopy-based techniques that evaluate a relatively limited number of cells and may be influenced by observer-dependent interpretation and sampling variability. In contrast, fluorescence-activated cell sorting (FACS) enables quantitative assessment of thousands of individual cells and provides objective measurement of HLA-G-positive cell populations at the single-cell level [37,38]. As a result, subtle differences in trophoblast abundance may be more readily detected by FACS than by conventional microscopic methods. These findings suggest that differences in analytical methodology may influence the apparent characterization of EVT populations. Whether subtle gestational age-related differences exist will require validation using standardized cohort-wide quantitative analyses. The present findings highlight the importance of analytical methodology when evaluating trophoblast recovery and suggest that flow cytometric approaches may provide greater sensitivity for detecting quantitative differences in EVT populations.

### 3.2. Clinical Implications and Future Applications of TRIC

Our results should also be interpreted in the context of previous TRIC studies. Earlier investigations primarily focused on demonstrating the feasibility of trophoblast isolation and optimizing recovery techniques using HLA-G-based immunomagnetic separation [8,10,30]. These studies established that EVT cells can be recovered between approximately 5 and 20 weeks of gestation with relatively high purity. The β-hCG positivity observed in the present study (63–73%) was lower than the high trophoblast purity, exceeding 90% in some reports, described in previous TRIC studies [10,22]. However, direct comparison of these values should be interpreted with caution because of differences in analytical strategies. Earlier studies often assessed highly enriched trophoblast populations following HLA-G-based isolation and, in some cases, further confirmation using fetal-specific genetic markers, whereas the present study quantified marker-positive cells within the broader recovered cell population. Accordingly, the lower β-hCG positivity observed in our study may partly reflect differences in the denominator and analytical approach rather than reduced isolation efficiency alone. Nevertheless, methodological factors related to immunomagnetic isolation, including antibody characteristics, bead conjugation, and washing conditions, may also contribute to variability in trophoblast enrichment across studies. However, relatively little attention has been directed toward understanding how EVT recovery changes throughout gestation. Furthermore, previous reports have yielded inconsistent findings regarding the influence of gestational age on trophoblast yield. Differences in study design, sample size, antibody selection, cell isolation methods, and analytical platforms may all contribute to variability among studies. In particular, most previous investigations relied primarily on immunocytochemistry or FISH-based assessments, whereas our study incorporated FACS-based quantification of HLA-G-positive cells. The increased analytical sensitivity of FACS may have enabled the detection of subtle differences in HLA-G-positive cell populations that were not apparent using conventional microscopy-based methods.

The potential implications of these findings extend beyond a characterization of gestational age-related recovery patterns. Because TRIC provides access to intact fetal-derived trophoblast cells containing the complete fetal genome, the quantity and quality of recovered EVT cells directly influence the success of downstream molecular analyses. Applications such as chromosomal analysis, fetal genotyping, next-generation sequencing, and investigation of monogenic disorders all depend on adequate trophoblast recovery. Consequently, improved characterization of EVT recovery across early gestation may enhance sampling strategies and facilitate future molecular diagnostic applications. In addition, TRIC-derived trophoblast cells have been increasingly utilized for investigating placental function and identifying biomarkers associated with adverse pregnancy outcomes. Improved understanding of EVT recovery dynamics may therefore contribute to the development of future approaches for early prediction of placental dysfunction.

Taken together, the principal contribution of the present study is the systematic multi-method assessment of TRIC-based EVT recovery across early gestation. Rather than establishing a definitive gestational age-dependent sampling window, the present findings demonstrate that TRIC enables the consistent recovery of fetal-derived trophoblast cells from early pregnancy while highlighting methodological considerations relevant to future studies. Beyond these findings, the present study also provides broader methodological and translational significance. Because relatively few research groups worldwide continue to perform systematic TRIC research, independent validation using complementary analytical approaches is essential for establishing the reproducibility and robustness of this technology. Furthermore, unlike conventional cell-free DNA-based NIPT, which is typically performed from approximately 9–10 weeks of gestation, TRIC enables the recovery of intact fetal-derived trophoblast cells from as early as 5 weeks of gestation. This unique capability provides access to the complete fetal genome during a developmental stage that is not readily accessible using current routine non-invasive prenatal testing. To the best of our knowledge, the present study is also among the very few to incorporate FACS together with immunofluorescence and FISH for evaluating TRIC-derived trophoblast cells within the same study, providing methodological insights that may facilitate future optimization of TRIC-based molecular and prenatal diagnostic applications.

Several limitations of this study should be acknowledged. First, the sample size within individual gestational age subgroups was relatively modest, which may have limited statistical power to detect differences using immunofluorescence and FISH analyses. Second, the reported cell numbers represent absolute recovered counts rather than values normalized to total input cell numbers. Therefore, variability in sampling efficiency, including differences in brushing efficiency, mucus content, and operator-dependent collection, may have contributed to the observed differences among groups. HLA-G-positive cell counts should be interpreted as descriptive observations rather than quantitative measures of gestational-age-related trophoblast recovery efficiency. Third, FISH analysis was restricted to pregnancies in which fetal cells could be unequivocally identified using Y-chromosome signals or fetal-specific chromosomal aneuploidies, resulting in a selected subset that may not be representative of the general pregnant population. Differences in placental development or trophoblast biology, particularly in aneuploid pregnancies, may have influenced the observed fetal-cell proportions and therefore may limit their generalizability. Accordingly, the FISH findings should be interpreted as confirmation of the fetal origin of the isolated trophoblast cells and evidence supporting the feasibility of fetal trophoblast recovery following TRIC, rather than as a population-level measure of gestational-age-related recovery efficiency. Fourth, FACS analysis was performed on selected samples and therefore should be interpreted as exploratory rather than definitive evidence of gestational age-related changes in trophoblast recovery. Fifth, the study evaluated trophoblast recovery metrics rather than downstream diagnostic performance, and therefore no conclusions can be drawn regarding the optimal gestational age for specific molecular applications. Finally, because TRIC samples originate from placental trophoblasts, the potential influence of placental heterogeneity and confined placental mosaicism cannot be completely excluded.

## 4. Materials and Methods

### 4.1. Patient Information

Cervical samples were collected from pregnant women who voluntarily participated in this study. Inclusion criteria included singleton intrauterine pregnancies between 5 and 19 weeks of gestation. A total of 53 pregnant women were included in the study. For FISH analysis, a selected subset of pregnancies with male fetuses or confirmed chromosomal aneuploidies (trisomy 13, 18, or 21) was analyzed to enable unequivocal identification of fetal cells. Participants were divided into three gestational-age groups (5–7 weeks, 8–12 weeks, and 13–19 weeks) to investigate the gestational period for trophoblast detection (see Table 1 and Supplementary Table S1). The study was approved by the Institutional Review Board of Gangnam Cha Medical Center (GCI-17-38), and written informed consent was obtained from all participants.

### 4.2. Endocervical Sampling

Endocervical samples were collected with the patient in the lithotomy position using a cytobrush inserted into the cervix with the aid of a speculum. The cytobrush was rotated 360° and advanced approximately 2 cm into the external cervix to obtain cervical cell samples. Collected samples were immediately transferred into Hanks’ Balanced Salt Solution (HBSS) (Gibco, Grand Island, NY, USA, #14170-112) and transported to the laboratory. The cells were centrifuged at 1000 rpm for 5 min at 4 °C and washed with cold phosphate-buffered saline (PBS) (Welgene, Gyeongsan-si, Gyeongsangbuk-do, Republic of Korea, #LB001-02) to remove mucus. For fixation, cells were incubated with 4% paraformaldehyde solution (4% PFA) (Biosolution Co., Suwon-si, Gyeonggi-do, Republic of Korea, #BP031-1L) for 10 min at 4 °C. After fixation, the supernatant was removed, and the cells were washed with cold PBS. Washed cells were stored at 4 °C prior to cell counting.

### 4.3. Immunomagnetic Isolation of Trophoblast Cells

Magnetic nanoparticles (250 nm) conjugated with goat anti-mouse IgG antibody (Clemente Associates, Prescott, AZ, USA) were incubated overnight at 4 °C with mouse anti-HLA-G antibody (10 µg/mL; clone 4H84, BD Biosciences, Franklin Lakes, NJ, USA, #557577). The next day, nanoparticles were washed with cold PBS using a magnetic stand (Thermo Scientific, Waltham, MA, USA) to remove excess antibody. Endocervical cells were resuspended in 1% bovine serum albumin (BSA) (Bovostar, Melbourne, VIC, Australia, #BSAS-NZ) and incubated overnight with anti-HLA-G conjugated nanoparticles. After magnetic immobilization, cells were separated into HLA-G positive and HLA-G negative cells. HLA-G positive cells were regarded as trophoblast-enriched population, whereas HLA-G negative cells were depleted of trophoblast cells and primarily composed of maternal cells.

### 4.4. Immunofluorescence

Isolated HLA-G positive cells were centrifuged at 1500 rpm for 5 min and loaded onto glass slides at 100 cells per slide using a Cytospin 7620 centrifuge (Wescor Inc., Logan, UT, USA). Cells attached to slides were blocked with 3% BSA for 1 h at 4 °C. Slides were incubated overnight at 4 °C with primary anti-β-hCG antibody (10 μg/mL; clone 5H4-E2, Thermo Scientific, Waltham, MA, USA). After washing with PBS, slides were incubated with Alexa Fluor^®^ 555 goat anti-mouse IgG secondary antibody (5 μg/mL; Invitrogen, Carlsbad, CA, USA, #A32727) for 1 h at room temperature. Nuclei were counterstained with 4′,6-diamidino-2-phenylindole dihydrochloride (DAPI; 1 μg/mL) (Invitrogen, Carlsbad, CA, USA, #D3571) for 10 min at room temperature. Mounted slides were observed using an Axio Imager 2 fluorescence microscope (Carl Zeiss, Oberkochen, Germany). Negative controls were performed using the same staining protocol without the primary antibody, and no specific fluorescence signal was detected. β-hCG-positive cells were identified based on background-corrected fluorescence intensity and expressed as a percentage of the total number of cells.

### 4.5. Fluorescence In Situ Hybridization (FISH)

FISH analysis included sample with confirmed fetal sex (male fetuses) or chromosomal aneuploidies. FISH was performed using chromosome 21 (q22.13–q22.2) probes labeled with Spectrum Orange (Abbott Molecular, Des Plaines, IL, USA, #08L54-020), chromosome X (Xp11.1–q11.1) probes labeled with Spectrum Orange (Abbott Molecular, Des Plaines, IL, USA, #32-110023), and chromosome Y (Yq12) probes labeled with Spectrum Green (Abbott Molecular, Des Plaines, IL, USA, #05J10-024). Hybridized slides were analyzed using an Axio Imager A2 fluorescence microscope (Carl Zeiss, Oberkochen, Germany) and the Isis FISH imaging system (MetaSystems Group Inc., Altlussheim, Germany). At least 10 cells showing probe signals were counted per slide.

### 4.6. Fluorescence-Activated Cell Sorting (FACS)

Cervical fluid samples were washed with PBS and cold staining buffer containing fetal bovine serum (FBS; BD Biosciences, San Jose, CA, USA, #554656). Cells were centrifuged at 1000 rpm for 5 min and resuspended in staining buffer containing Human BD Fc Block™ (BD Biosciences, San Jose, CA, USA, #564220) for 10 min at 4 °C. Cells were incubated with primary anti-HLA-G antibody and anti-CD45 antibody (Abcam, Cambridge, UK, #ab8216), each diluted 1:100 in staining buffer, for 20 min, followed by washing with staining buffer. Alexa Fluor^®^ 488 goat anti-mouse IgG secondary antibody (5 μg/mL; Invitrogen, Carlsbad, CA, USA, #A11034) diluted 1:200 was then added and incubated for 20 min in the dark. After washing, stained samples were analyzed within 4 h using a CytoFLEX flow cytometer (Beckman Coulter, Brea, CA, USA). Data were analyzed using CytExpert software version 2.4 (Beckman Coulter, Brea, CA, USA). Target cells were initially selected based on forward and side scatter (FSC/SSC) characteristics to exclude debris. Maternal leukocytes were excluded by gating on CD45-negative cells, and extravillous trophoblasts were subsequently identified as CD45^−^/HLA-G^+^ cells. Gating thresholds were established using isotype control samples. HLA-G-positive cells expressing green fluorescence in the FITC channel were considered trophoblast-enriched populations.

### 4.7. Statistical Analyses

Statistical analyses were performed using R version 4.0.2 (R Foundation for Statistical Computing, Vienna, Austria). For samples analyzed in technical replicates, replicate measurements were averaged to obtain a single value for each participant before statistical analysis. Cell counts and expression levels were expressed as mean ± standard deviation (SD), whereas graphical error bars represented the standard error of the mean (SE). Comparisons among gestational-age groups were performed using one-way analysis of variance (ANOVA), followed by Tukey’s honestly significant difference (HSD) test for multiple comparisons. A *p*-value < 0.05 was considered statistically significant.

## 5. Conclusions

Although TRIC has considerable potential for prenatal diagnosis and placental research, relatively few studies have systematically evaluated fetal trophoblast recovery across early gestation using complementary analytical approaches. Our findings support the feasibility and relative stability of TRIC-based fetal trophoblast recovery across early gestation, as demonstrated by immunofluorescence and FISH analyses. Furthermore, fetal-derived EVT cells were successfully recovered from pregnancies as early as 5 weeks of gestation, further supporting the feasibility of TRIC during early pregnancy. Further studies using larger cohorts and standardized quantitative approaches are warranted to more precisely characterize EVT recovery across early gestation and to further refine TRIC-based molecular and diagnostic applications.

## Figures and Tables

**Table 1:** Clinical Characteristics of the Study Population.

Group		5 w–7 w (N = 18)	8 w–12 w (N = 15)	13 w–19 w (N = 8)
Maternal Age		35.0 ± 4.2 ^a^	33.1 ± 4.4 ^a^	35.0 ± 3.2 ^a^
Gestational Age (day)		49.6 ± 5.0 ^a^	66.4 ± 11.2 ^b^	117.1 ± 12.7 ^c^
Gravidity	1	7 (38.9%)	10 (66.7%)	3 (37.5%)
	2	9 (50.0%)	2 (13.3%)	4 (50.0%)
	3	2 (11.1%)	2 (13.3%)	0 (0.0%)
	4	0 (0.0%)	1 (6.7%)	1 (12.5%)
Parity	0	9 (50.0%)	10 (66.7%)	4 (50.0%)
	1	8 (44.4%)	5 (33.3%)	4 (50.0%)
	2	1 (5.6%)	0 (0.0%)	0 (0.0%)
BMI		22.0 ± 4.1 ^a^	21.0 ± 3.0 ^a^	21.7 ± 2.7 ^a^

^a–c^ Different superscript lowercase letters indicate statistically significant differences among groups (*p* < 0.05).

**Table 2:** Immunofluorescence-based assessment of isolated trophoblast cells according to gestational age.

Group	Age	Gestational Age (day)	Total Endocervical Cell	HLA-G Positive	β-hCG
**5 w–7 w (N = 18)**	35.0 ± 4.2	49.6 ± 5.0	7.69 × 10^5^ ± 8.9 × 10^5^	1846.8 ± 1578.8 ^a^	63.8 ± 18.5 ^a^
**8 w–12 w (N = 15)**	33.1 ± 4.4	66.4 ± 11.2	8.82 × 10^5^ ± 7.6 × 10^5^	5559.1 ± 6347.6 ^b^	64.7 ± 17.3 ^a^
**13 w–19 w (N = 8)**	35.0 ± 3.2	117.1 ± 12.7	8.50 × 10^5^ ± 1.1 × 10^5^	2049.5 ± 2203.1 ^a^	73.1 ± 5.9 ^a^

^a, b^ Different superscript lowercase letters indicate statistically significant differences among groups (*p* < 0.05).

**Table 3:** FISH-based assessment of fetal cell proportions according to gestational age.

Group	Age	Gestational Age (day)	Total Endocervical Cell	HLA-G Positive	FISH (%)
**5 w–7 w (N** **= 2)**	33.5 ± 2.1	52.0 ± 2.8 ^a^	30.3 × 10^5^ ± 6.4 × 10^5^	2475 ± 318.2 ^a^	18.3 ± 6.79 ^a^
**8 w–12 w (N = 12)**	34.3 ± 5.5	78.1 ± 10.7 ^b^	9.0 × 10^5^ ± 8.5 × 10^5^	4106.4 ± 6589.3 ^a^	18.1 ± 7.05 ^a^
**13 w–19 w (N = 6)**	37.5 ± 3.5	113.8 ± 16.0 ^c^	3.4 × 10^5^ ± 2.0 × 10^5^	8553 ± 18,353.9 ^a^	20.2 ± 3.22 ^a^

^a–c^ Different superscript lowercase letters indicate statistically significant differences among groups (*p* < 0.05).

## Data Availability

The original contributions presented in this study are included in the article/Supplementary Materials. Further inquiries can be directed to the corresponding author.

## Supplementary Materials

The following supporting information can be downloaded at: https://www.mdpi.com/article/10.3390/ijms27156641/s1.
