# Supplementary material for: Trophoblast Retrieval from the Cervix (TRIC) Across Early Gestation: A Multi-Method Assessment of Extravillous Trophoblast Recovery
Source: Int J Mol Sci. 2026 Jul 25;27(15):6641. doi: 10.3390/ijms27156641 (PMC13465896; doi:10.3390/ijms27156641)
Supplement: Supplementary file 1 [file ijms-27-06641-s001.zip › ijms-4387947-supplementary.pdf]

Supplementary Materials

Supplementary Figure S1. Representative FACS gating strategy and corresponding isotype controls for the identification of CD45−/HLA-G+ trophoblast cell populations. (A) gestational age 5 weeks 5 days; (B) gestational age 8 weeks 0 days.

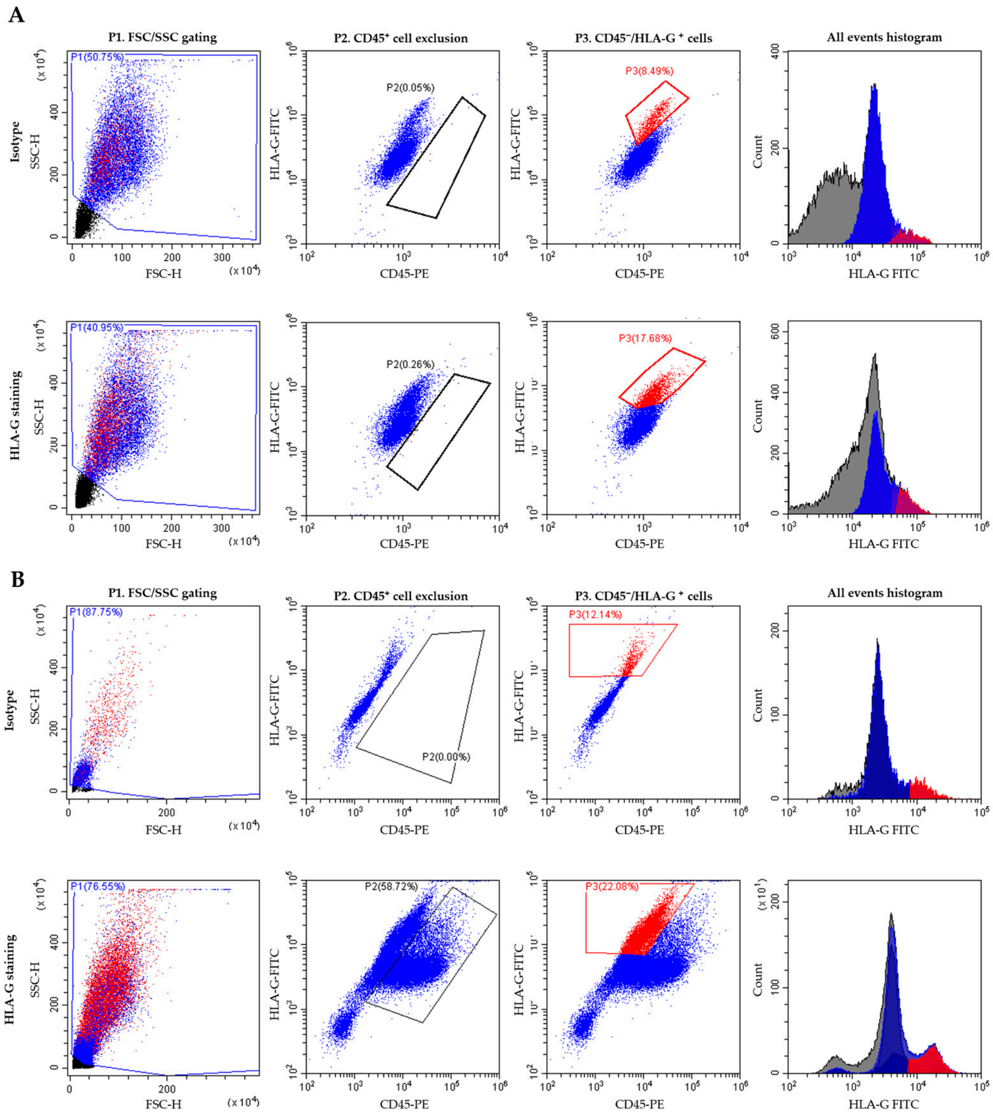

Supplementary Table S1. Individual clinical characteristics of study participants.

Group	NO.	Maternal Age	Fetal Sex	Gestational Age(day)	gravidity	parity	BMI
5 w-7 w	1	36	M	51	3	2	21.72
	2	36	M	36	2	1	16.36
	3	38	F	55	2	0	34.29
	4	36	M	47	2	1	19.14
	5	40	N/A	44	1	0	23.05
	6	40	F	47	2	1	25.82
	7	35	M	54	2	1	25.44
	8	32	M	50	2	1	22.21
	9	30	M	50	3	1	19.83
	10	41	F	54	1	0	19.47
	11	25	F	47	1	0	21.11
	12	35	F	54	2	1	24.22
	13	35	F	48	2	1	20.45
	14	38	F	53	1	0	19.47
	15	30	F	55	1	0	17.44
	16	37	F	54	1	0	23.95
	17	30	M	44	1	0	17.63
	18	36	M	50	2	0	23.71
8 w-12 w	1	27	M	58	2	1	24.84
	2	34	M	77	1	0	20.03
	3	40	F	58	4	1	21.22
	4	33	M	80	1	0	26.30
	5	36	M	72	1	0	23.03
	6	27	M	87	1	0	15.94
	7	37	F	57	3	1	25.65
	8	27	M	57	1	0	21.23
	9	29	M	58	1	0	19.68
	10	36	M	62	3	1	19.00
	11	32	M	57	1	0	20.76
	12	36	F	63	2	1	21.22
	13	30	F	68	1	0	22.02
	14	33	F	56	1	0	17.15
	15	40	M	86	1	0	17.51
13 w-19 w	1	34	M	117	1	0	19.63
	2	35	F	119	2	1	22.68
	3	39	N/A	133	4	1	23.95
	4	37	M	135	1	0	21.19
	5	36	M	112	2	1	20.42
	6	30	M	112	2	0	26.92
	7	31	M	114	2	1	19.53
	8	38	F	95	1	0	19.23

Supplementary Table S2. Individual immunofluorescence data used for β-hCG–positive cell quantification.

Group	NO.	Maternal Age	fetal sex	Gestational Age (day)	Total endocervical cell	HLA-G positive	β-hCG
5 w-7 w	1	36	M	51	$4.6X10^5$	3,650	67
	2	36	M	36	$4.75X10^4$	2,750	70.3
	3	38	F	55	$1.4X10^5$	1,500	50.0
	4	36	M	47	$6.45X10^5$	3,000	63.0
	5	40	N/A	44	$8.75X10^4$	100	29.2
	6	40	F	47	$1.4X10^5$	100	84.0
	7	35	M	54	$3.48X10^6$	2,250	88.0
	8	32	M	50	$2.58X10^6$	1784	80.7
	9	30	M	50	$8.5X10^5$	1,875	66.2
	10	41	F	54	$7.45X10^5$	900	51.7
	11	25	F	47	$8.7X10^5$	375	86.7
	12	35	F	54	$2.4X10^5$	6,500	45.9
	13	35	F	48	$2.5X10^5$	275	79.3
	14	38	F	53	$1.11X10^6$	1,475	34.6
	15	30	F	55	$3.4x10^5$	625	84.6
	16	37	F	54	$8.8x10^5$	2,750	50.0
	17	30	M	44	$2.0X10^5$	2,333	45.5
	18	36	M	50	$3.28X10^5$	1,000	71.7
8 w-12 w	1	27	M	58	$6.0X10^4$	1,000	29.1
	2	34	M	77	$1.6X10^6$	575	96.0
	3	40	F	58	$8.8x10^5$	17,875	64.5
	4	33	M	80	$1.6X10^6$	18,625	54.1
	5	36	M	72	$5.89 X 10^5$	5,750	46.7
	6	27	M	87	$5.35 X 10^5$	4,250	76.1
	7	37	F	57	$5.1X10^5$	300	77.0
	8	27	M	57	$2.23X10^6$	9,000	53.4
	9	29	M	58	$3.25X10^5$	1,000	78.0
	10	36	M	62	$1.06X10^6$	12,525	69.1
	11	32	M	57	$1.8X10^5$	1,500	53.4
	12	36	F	63	$5.7X10^5$	8,000	60.4
	13	30	F	68	$1.62X10^5$	375	85.7
	14	33	F	56	$2.55x10^5$	1,250	51.8
	15	40	M	86	$2.48X10^6$	1,361	75.9
13 w-19 w	1	34	M	117	$4.5X10^5$	1,750	78.0
	2	35	F	119	$4.6X10^5$	1,000	78.0
	3	39	N/A	133	$3.7X10^5$	125	81.8
	4	37	M	135	$5.71X10^3$	1,075	66.7
	5	36	M	112	$5.7X10^5$	2,333	71.7
	6	30	M	112	$7.1X10^5$	1,778	74.2
	7	31	M	114	$3.45X10^6$	1,085	68.6
	8	38	F	95	$2.15X10^5$	7,250	65.7

Supplementary Table S3. Individual FISH data used for fetal cell proportion analysis.

Group	NO	Age	Fetal sex	Karyotype	Gestational Age (day)	Total endocervical cell	HLA-G positive	FISH
5 w-7 w	1	32	M	46, XY	50	$2.58X10^6$	2700	13.5
	2	35	M	46, XY	54	$3.48X10^6$	2,250	23.1
8 w-12 w	1	27	M	46, XY	57	$2.23X10^6$	9,000	18.2
	2	29	M	46, XY	58	$3.25X10^5$	1,000	10.6
	3	36	M	46, XY	72	$5.89X10^5$	5750	11.8
	4	34	M	46, XY	77	$1.60X10^6$	575	29.4
	5	33	M	46, XY	80	$1.60X10^6$	23,250	10.6
	6	42	M	46, XY+13	81	$3.00X10^4$	1,000	23.1
	7	34	M	46, XY+21	82	$2.55X10^4$	250	14.3
	8	29	M	46, XY	83	$6.40X10^5$	1,050	21.8
	9	42	F	46, XY+21	84	$4.55X10^5$	500	20.0
	10	40	M	46, XY	86	$2.48X10^6$	1,277	28.9
	11	27	M	46, XY	87	$5.35X 10^5$	4,250	8.6
	12	38	M	46, XY+18	90	$3.50X10^5$	1,375	20.0
13 w-19 w	1	38	M	46, XY+21	93	$1.48X10^5$	750	15.8
	2	38	M	46, XY+21	99	$5.30X10^5$	900	24.0
	3	42	F	46, XY+21	112	$4.20X10^5$	1,670	20.0
	4	34	M	46, XY+21	117	$4.50X10^5$	1,750	19.4
	5	33	F	47, XXX	128	$4.80X10^5$	46,000	23.8
	6	40	M	46, XY+21	134	$3.75X10^4$	250	18.2
